# Transactions of the American Medical Association, Vol. IX. 1856

**Published:** 1857-06

**Authors:** 


*Transactions of the American Medical Association, Vol. IX.* 1856. Philadelphia: Printed for the Association by T. R. & P. G. Collins.

We had intended to continue our notice of the different papers in this volume, but our present time and space will permit only an enumeration of those not heretofore noticed. They are as follows: 'Topography of the Eastern Shore of Maryland.' 'Report on the Yellow Fever of Charleston in 1854,' by Dr. Cain. 'Report on the Epidemics of Louisiana, Mississippi, Arkansas, and Texas,' by Dr. Fenner. 'Report on the Sanitary Condition, Meteorology, and Mortality of New Orleans, in 1854–55,' by Dr. Barton. 'Report on Strychnine,' by Dr. L. H. Steiner; and the 'Prize Essay on the Arterial Circulation,' by Dr. Henry Hartshorne, of Philadelphia.

All these are papers of interest and value, and we again express the hope that the volume may be purchased and read by a large portion of the physicians of the North-West.

